# Biobank-scale genotype similarity search and dynamic patient-matched cohort creation with GenoSiS

**DOI:** 10.1101/gr.280278.124

**Published:** 2026-08

**Authors:** Kristen Schneider, Murad Chowdhury, Mariano Tepper, Jawad B. Khan, Jonathan A. Shortt, Chris Gignoux, Ryan M. Layer

**Affiliations:** 1 Computer Science Department, University of Colorado, Boulder, Colorado 80309, USA;; 2 BioFrontiers Institute, University of Colorado, Boulder, Colorado 80303, USA;; 3 Intel Labs, Hillsboro, Oregon 97124, USA;; 4 Colorado Center for Personalized Medicine, University of Colorado, Anschutz Medical Campus, Aurora, Colorado 80045, USA;; 5 Department of Biomedical Informatics, University of Colorado, Anschutz Medical Campus, Aurora, Colorado 80045, USA

## Abstract

Many patients do not experience optimal benefits from medical advances because clinical research does not adequately represent them. Although the diversity of biomedical research cohorts is improving, ensuring that individual patients are adequately represented remains challenging. We propose a new approach, GenoSiS, which leverages machine learning–based similarity search to dynamically find patient-matched cohorts across different populations quickly. These cohorts could serve as reference cohorts to improve a range of clinical analyses, including disease risk score calculations and dosage decisions. Although GenoSiS focuses on finding genetic similarity within a biobank, our similarity search architecture can be extended to represent other medically relevant patient characteristics and search other biobanks.

Healthcare providers make treatment decisions by blending their experience, patient concerns, and data from clinical trials. Although clinical trials are the gold standard for determining the safety and efficacy of medical interventions, health disparities arise, in part, because it is challenging to apply the results from a study to patients who are not represented by the subjects within the study cohort. Ding et al. (2023) quantified the genetic component of this effect by showing that the more distant an individual is from samples in the reference cohort, the less accurate their predicted disease risk was. Their results also showed that the straightforward approach of replicating a study for different groups (e.g., by genetic ancestry) provides only a marginal improvement for those who fit neatly into a group and no benefit for those who do not.

Mitigating health inequalities resulting from insufficient representation in clinical trial cohorts may necessitate creating reference cohorts tailored to individual patients. These patient-matched cohorts could, for example, be used to replicate clinical studies to generate evidence that inform representative treatment recommendations for all patients, especially those from populations that have traditionally been marginalized in healthcare research. Although university biobanks are approaching the size and diversity required to fulfill many patients’ needs, traditional indexing and exact search methods do not scale. We propose leveraging machine learning–based approximate similarity search architectures to find cohorts of similar individuals dynamically.

Similarity search architectures find the nearest neighbors of an object using numerical vectors called embeddings. Each object is represented with an embedding, and objects with similar attributes have embeddings that are close in the vector space. A common approach to map objects to embeddings is to use a neural network trained with a corpus of objects with known distances. Although embeddings are highly general and have been used for objects from words and video to DNA sequences, the primary challenge is determining the most relevant features. For diagnosis and treatment decisions, patient similarity is complex and context dependent. Here, we start with genetic similarity because it is stable, and genetic distance is a well-researched topic, but in the future, patient similarity will likely include other more dynamic data types, including methylation, expression, and health records, all of which can be represented as embeddings and can be explored using a similarity search database. Alternatively, structured fields can be incorporated to the search using hybrid techniques that include attribute filtering.

Here, we present genotype similarity search (GenoSiS), an architecture that combines a learned haplotype embedding with approximate nearest-neighbor (ANN) indexing to construct patient-matched cohorts at biobank scale. We aim to (1) introduce the embedding model and search architecture; (2) validate cohort quality across closely related individuals (deCODE pedigrees), distantly related individuals spanning global populations (The 1000 Genomes Project [1KGP]), and a clinical biobank of tens of thousands of samples (Colorado Center for Personalized Medicine's [CCPM]); and (3) examine cohort quality when the query population is underrepresented in the database, as well as identify potential inequities introduced by the embedding pipeline.

## Results

**Figure 1.**
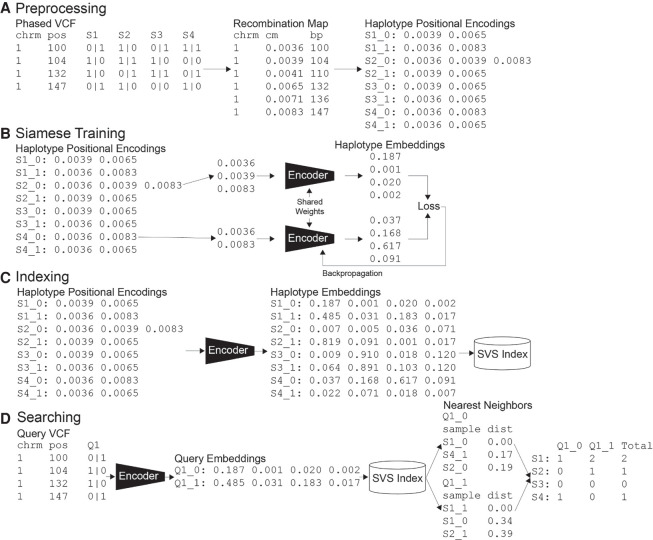
The genotype similarity search (GenoSiS) workflow. (*A*) Starting with a VCF and recombination map, GenoSiS generates a haplotype positional encoding vector for each sample. (*B*) We then train an embedding model to minimize the difference between encoding and embedding distances. (*C*) Using the trained model, we create embeddings for all sample haplotypes and index them with Intel's SVS. (*D*) The index is queried using a sample's embeddings to find its nearest neighbors, and those results are aggregated to determine the sample's cohort.

GenoSiS uses a novel genotype embedding model and the Intel scalable vector search (SVS) performance library (Aguerrebere et al. 2023) to find representative cohorts quickly for any patient. First, we produce positional encodings for each sample's haplotype from the phased genotypes in a variant call file (VCF) (Danecek et al. 2011) and a recombination map (Fig. 1A; Browning et al. 2021). Next, using a Siamese neural network (Bromley et al. 1993), we learn genetic embeddings from pairs of haplotype encodings (Fig. 1B) and then produce embeddings for all haplotypes with the trained model and index them using SVS (Fig. 1C). Using a sample's genotypes as a query, GenoSiS can find the nearest neighbors with SVS, from which it constructs a sample's matched cohort (Fig. 1D).

GenoSiS quickly and accurately found high-quality matched cohorts across data sets and populations. In less than a second, GenoSiS identified representative cohorts among families in deCODE pedigrees, across populations among the 3201 samples from the 1000 Genomes Project (1KGP) (The 1000 Genomes Project Consortium et al. 2010, 2012, 2015; Fairley et al. 2020) and in the 73,346 samples from the CCPM biobank (Wiley et al. 2024). GenoSiS is also highly general. With an embedding model trained on genotypes from whole-genome sequencing (WGS), GenoSiS accurately constructed matched cohorts in CCPM data, which used targeted sequencing and imputation. These results demonstrate that GenoSiS is a robust method that has the potential to significantly improve patient representation in healthcare.

### Cohort genetic similarity

**Figure 2.**
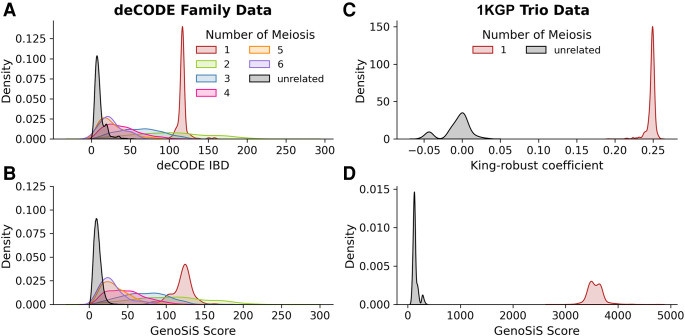
Genetic distance distributions among closely related samples. (*A*,*B*) deCODE IBD (*A*) and GenoSiS score (*B*) distributions for deCODE families. (*C*,*D*) King-robust coefficient (*C*) and GenoSiS score (*D*) distributions for 1KGP trios. The deCODE IDB and GenoSiS score ranges correspond to the number of centimorgans considered, whereas the King-robust coefficient is normalized such that first-degree relatives have a score of about 0.25, second-degree relatives a score of about 0.125, and so forth. (*A*,*B*) Two haplotypes for Chromosome 18. (*C*,*D*) All autosomes. Distributions plotted and colored by numbers of meiotic events separating two samples in which the number of meiotic events corresponds to family relationships (e.g., 1 = parent/child, 2 = grandparent/grandchild, full-siblings, etc.). All cohorts were size 20.

Genetic similarity can seem straightforward. We are most related to our parents and children, followed by siblings, grandparents, and so on. Outside our family, we are most related to those with similar geographic ancestry. Quantifying genetic similarity is a complex and active area of research (Suarez-Pajes et al. 2021), and when we compare nominal and quantitative genetic similarity, the distributions can be broad, overlapping, and potentially contradictory (Fig. 2). With this uncertainty, there is no single genetic similarity ground truth from which we can measure accuracy and precision. To determine how well GenoSiS's cohorts relate to the query sample, we compared our result to other widely used methods at different scales of relatedness and in different populations.

#### Closely related individuals

We start by considering relatedness among biological family members because we have an expectation of how related two people are based on the number of meiotic events which separate them. On average, ∼50% of our autosomal DNA passes to our children, 25% to our grandchildren, and so on, but even at this scale, the randomness of recombination and other factors complicate these expectations. Full siblings can share <40% to >60% of their DNA, and cousins can share between 5% and 20% (Hill and Weir 2011; Henn et al. 2012).

To analyze performance among closely related individuals, we used 10 pedigrees (Chromosome 18) from deCODE Genetics, each spanning four to six generations. For each sample, we generate GenoSiS cohorts in which *k* = 20 and compare the GenoSiS's scores (see Methods) between pairs of samples to their nominal relationship and their deCODE IBD metric (Fig. 2A,B). Additionally, we compared the difference between the GenoSiS score and the King-robust kinship coefficient score among the 608 trios in the 1KGP cohort and between unrelated samples (Fig. 2C,D).

In the deCODE genetic pedigrees and the 1KGP trios, the GenoSiS score showed a correlation with sample relatedness that was consistent with other methods. For 10 deCODE pedigrees, each spanning four to six generations, the GeonoSiS score and deCODE's IBD metrics easily distinguished between parent/child relationships and unrelated samples. Although the distribution medians for more distant relationships decreased as the number of meiosis increased, as expected, there was significant overlap in the distributions, making reliable differentiation among these relationships difficult for either method (Fig. 2A,B). Similarly, the trios (i.e., mother, father, child) in the 1KGP were distinguished from unrelated samples using the GenoSiS score and King-robust coefficient (Fig. 2C,D). These results highlight the effectiveness of the GenoSiS score in quantifying genetic relatedness while also demonstrating the challenges inherent in differentiating more distant relationships.

#### Distantly related individuals

In this work, we shall define a distant relative as any individual with a degree of relatedness greater than four. Although our expectations among distantly related individuals are weaker, we are generally more related to those whose ancestors originated near our ancestors, this search is more relevant because biobank-derived cohorts are more likely to include nonfamily members. Given our relatedness expectations at this scale, we evaluated cohorts based on genetic ancestry specificity. Because of the complexity of human ancestry and population stratification, it is challenging to definitively state that a cohort should comprise *P*% of individuals from a specific group or that participants within a cohort should have a GenoSiS score of *S* or higher. For the analysis of distantly related samples, we can say that for a fixed cohort size (e.g., 20), a cohort with more individuals from the same population as the query sample (i.e., 14/20 are from the same population as the query) is preferred over one with more individuals from a different population than the query sample (i.e., 14/20 are from a different population than the query). Further discussion about the quality of these cohorts depends on the population's natural history, and some of this is addressed in the next subsection “Cohorts with poor representation” and elaborated on in the Discussion section.

To evaluate cohorts formed from distantly related samples, we considered 3202 1KGP samples, which included 26 populations that we refer to as subpopulations (e.g., Esan [ESN], Great Britain [GBR]) (for the full list, see Supplemental Table S1), each of which falls into one of five superpopulations (African [AFR], American [AMR], East Asian [EAS], European [EUR], and South Asian [SAS]). We retained all trios to mimic a real-world biobank for which we may want closely related samples to appear in cohorts when available. We removed one sample that is listed under both the AFR and EUR superpopulations. Although the GenoSiS operates the same for samples of mixed ancestry, we wanted to keep our analysis for the 1KGP data consistent for only samples with known, single ancestry. We used these data to compare GenoSiS to PLINK's DST and pi-hat metrics (Purcell et al. 2007) and the King-robust kinship coefficient estimator (Manichaikul et al. 2010). With GenoSiS, we queried individual samples and used their nearest neighbors to determine their unique custom cohorts for each individual. In contrast, the other three methods provide N × N pairwise relatedness scores. From these scoring matrices, we sorted the results for each sample and retained the top scores to form their custom cohort.

**Figure 3.**
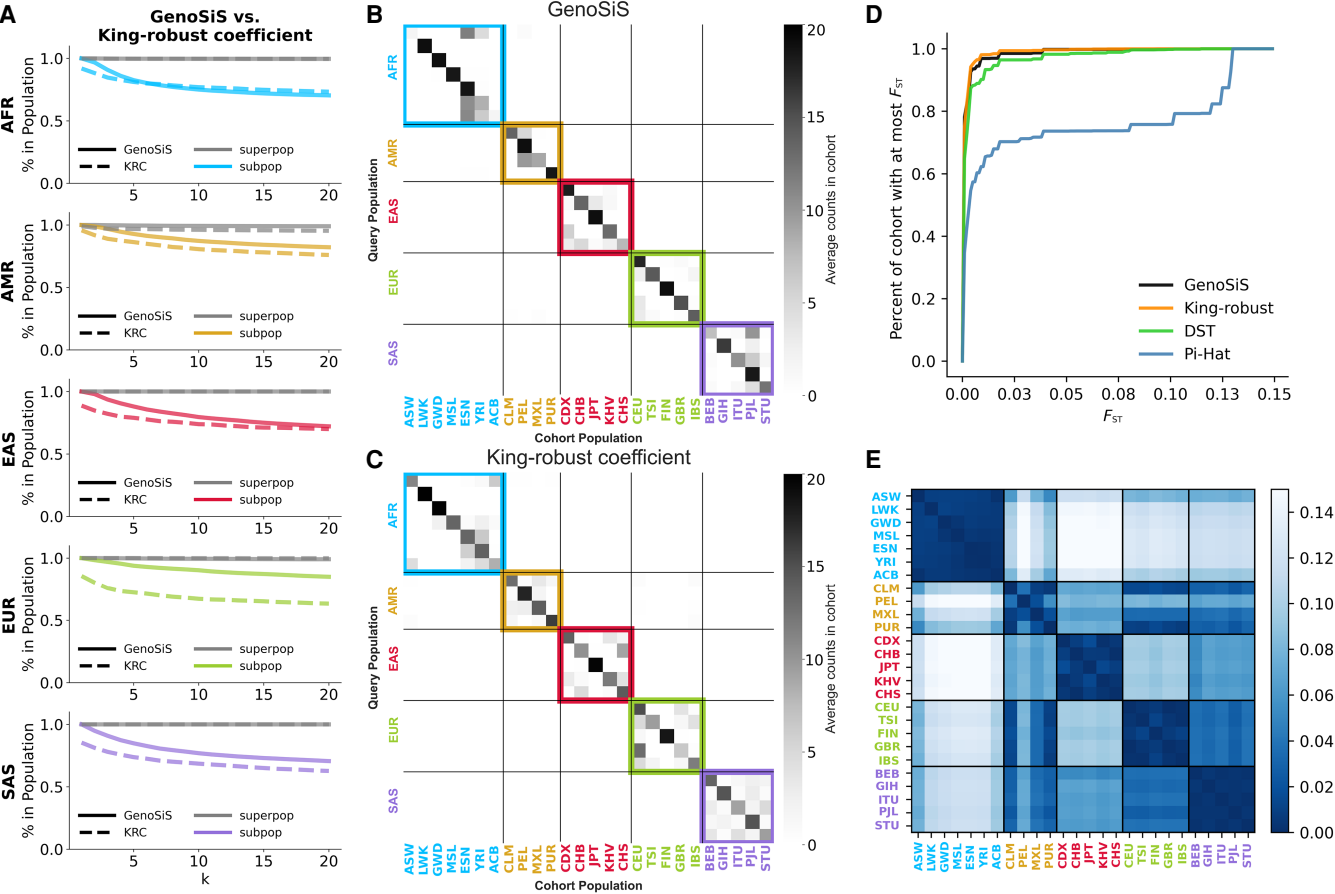
Analysis of 1KGP cohorts. (*A*) Percentage of samples from the 1KGP cohorts which are in the super- and subpopulations of the query samples as the size of the cohorts increases from one to 20. GenoSiS is plotted as a solid line, and the King-robust coefficient is plotted as a dashed line. Lines for superpopulations are plotted in gray, and lines for subpopulation are plotted in a color corresponding to the population. (*B*,*C*) Average subpopulation counts of appearances in cohorts generated by GenoSiS (*B*) and King-robust coefficient (*C*). For both *B* and *C*, superpopulation labels for query samples are listed on the vertical axis and are colored accordingly. Subpopulation labels for samples in the representative cohort are listed on the horizontal axis and are colored according to their respective superpopulation. *F*_ST_ is a statistical measure of genetic differentiation between populations (Privé et al. 2022). (*D*) Empirical distribution function tracking the percentage of samples identified by three comparison methods that were from subpopulations with an *F*_ST_ value less than equal to than a specified threshold. For instance, 78% of samples identified by GenoSiS were from populations with an *F*_ST_ ≤ 0.001, King-robust had 75.2%, DST had 64.8%, and pi-Hat had 34.6%. A lower *F*_ST_ and darker blue color indicates higher genetic similarity. (*E*) *F*_ST_ for all 1KGP subpopulations grouped by superpopulation. Cohort size *k* = 20 for all scenarios.

Cohorts drawn from fixed populations will be more likely to include more diverse samples as they grow larger. To track the specificity of cohorts as they become more diverse, we sorted cohorts in decreasing relatedness to the query sample (i.e., a cohort with one sample includes only the most related sample and one with 20 includes the full cohort) and tracked the proportion of the cohort that matched the query's super- and subpopulations (Fig. 3A). The 1KGP cohorts created by GenoSiS contained a higher density of samples matching the query at both the super- and subpopulation levels. Between 98.97% and 100% of samples in the GenoSiS cohorts matched the query sample's superpopulation, whereas the other three methods included up to 30% of samples from other superpopulations. In nearly every instance, the GenoSiS cohorts retained more samples with matching subpopulations and never included >20% of different subpopulations. The one exception was the African population, for which GenoSiS and the King-robust kinship coefficient retained subpopulation samples at about the same rate. For a comparison of GenoSiS with PLINK DST and pi-hat, see Supplemental Figure S1.

To understand the composition of the cohorts produced by each method at a more granular level, we organized the cohorts into groups based on the query sample's subpopulation and calculated the average occurrence of each subpopulation in each group (Fig. 3). For example, the GenoSiS African Ancestry in Southwest United States (ASW) cohorts (Fig. 3B) had samples from three different superpopulations (95.6% African, 4% European, 0.4% American) and eight different subpopulations. Among the eight subpopulations, three were dominant: Esan in Nigeria (ESN), Yoruba in Ibadan, Nigeria (YRI), and ASW, with an average of 11.3, 5.1, and 1.7 samples per cohort, respectively. This structure is consistent with our current understanding of African American genetic ancestry (Gouveia et al. 2020; Micheletti et al. 2020). For further discussion on differences between the similarity metrics of GenoSiS and Kinship, see Supplemental Note 1.

GenoSiS produced cohorts with more in-subpopulation samples. Four of the 26 GenoSiS subpopulation cohorts contained exclusively samples matching the queries’ subpopulations, which did not occur with other methods. Most (65%) of the GenoSiS subpopulation cohorts contained at least 70% of samples matching the queries’ subpopulation. In comparison, only 19.2%, 34.6%, and 42.3% of cohorts produced by Pi-hat, DST, and King-robust kinship methods, respectively, met this criterion. Overall, GenoSiS and King-robust produced cohorts with samples that were from highly similar subpopulations based on subpopulation to subpopulation *F*_ST_ (Fig. 3D,E; Privé et al. 2022).

#### Cohorts with poor representation

Identifying representative cohorts in a biobank relies on the biobank having individuals who are genetically similar to the patient. Because biobank diversity reflects regional diversity, health systems can expect to encounter patients who are poorly represented in their biobank. Therefore, it is crucial to determine if a patient's cohort is sufficiently representative.

**Figure 4.**
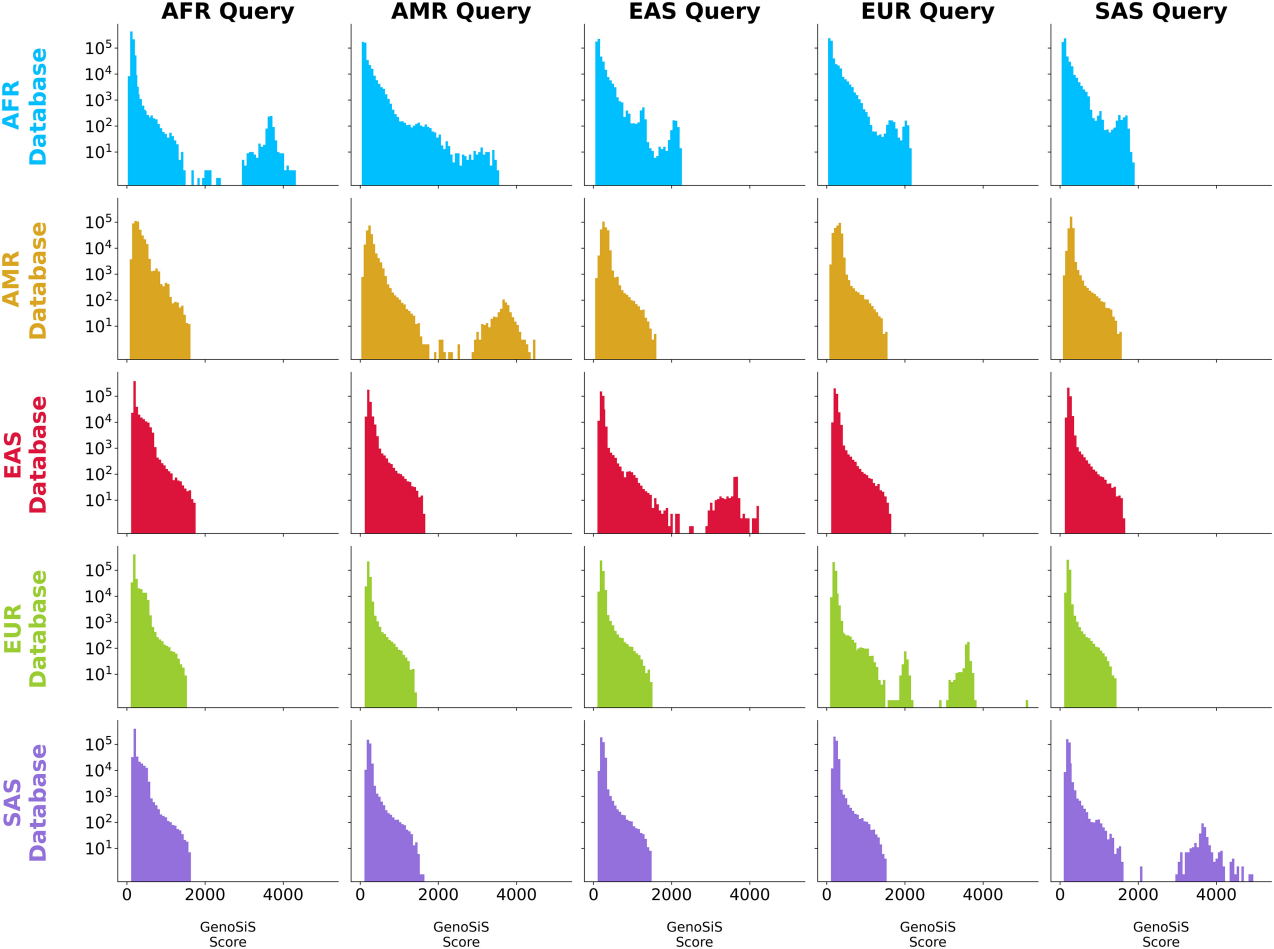
Cohort composition for queries with matched and mismatched 1KGP populations. Rows are representative and colored by the population of the database (i.e., samples which can appear in the GenoSiS cohort); columns are representative of the query population. In cases in which the query and database sets were from the same superpopulation (diagonal), we separately label when the cohort samples matched the query samples’ sub- and superpopulation labels.

To understand the dynamics of queries against underpowered databases, we used data from the 1KGP to construct six population-specific GenoSiS indexes and six population-specific query sets and then tracked the GenoSiS score for each query set against each index (Fig. 4). As expected, GenoSiS scores were lower when the index and query set diverged. We can establish thresholds from these results to determine if a cohort adequately represents the query. For example, using the 1KGP, we might exclude cohort members with a GenoSiS score below 1500 and assess whether the remaining members are sufficient for future analysis. Although exact threshold will be specific to each database and type of analysis, these results clearly demonstrate the need to assess cohort representation.

#### Potential inequities

Although GenoSiS performance is consistent across 1KGP populations, it is still critical to identify any potential inequities when training machine learning models. Here, we define an inequity as any instance in which performance differs across populations. These inequities often arise from inadequate or biased training strategies and can typically be resolved by improving the training corpus. When improved training cannot resolve the bias, it must be detected and understood, and steps must be taken to mitigate its effects.

**Figure 5.**
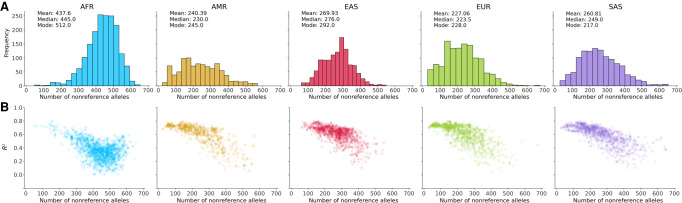
Embedding quality is worse for individuals with more nonreference alleles, who most often have African genetic ancestry. (*A*) The distribution of nonreference allele counts by population. (*B*) The relationship between a sample's embedding quality, measured by the correlation coefficient (*R*^2^) between the genotype encoding and embedding distances, and the number of nonreference alleles in the sample's genome.

For example, in the embedding stage, GenoSiS converts a sample's genotypes to a genetic embedding. Genotypes are then represented as two variable-length positional haplotype encodings with one entry for every nonreference allele, as well as two vectors per sample. Haplotype embeddings are a fixed-length floating point vector. The conversion of a variable-length vector to a fixed-length vector has the potential to introduce bias if different populations have different haplotype vector lengths. The manifestation of this potential bias comes from a reference bias effect in which individuals with African ancestry have more nonreference alleles and longer haplotype encodings (Fig. 5A).

To investigate this potential source of bias, we compared how the number of nonreference alleles in a sample corresponds to embedding quality. Our model learns embeddings by minimizing the difference between the distances of haplotype encoding pairs and the distances of the corresponding haplotype embedding pairs. We can measure the overall quality as the linear correlation (*R*^2^) between the genotype and embedding distances. Ideally, high correlations across populations would indicate no bias. However, the African population has a distinct decrease in *R*^2^ (Fig. 5B). Although this bias does not significantly affect our ability to find cohorts for this population, we plan to address this issue by systematically investigating which regions across subpopulations suffer from density-related performance degradation, expanding the training set to incorporate such examples and to explore various modifications to the training objective/methodology such as adding a segment density or entropy regularization term. For further discussion on the impact of population representation on embedding performance, see Supplemental Note 2.

#### Colorado center for personalized medicine Biobank

The most compelling application of GenoSiS is in searching biobanks for cohorts genetically similar to patients. This search is similar to searching the 1KGP, in which the cohorts are mostly nonfamily members. However, unlike the 1KGP, a biobank's population structure is determined by its participants, often reflecting the regional diversity where the samples were collected. For example, the CCPM biobank's (Wiley et al. 2024) diversity mirrors that of the broader Rocky Mountain region. Based on inferred genetic ancestry cluster memberships, the biobank consists of 80% European ancestry, 10% Hispanic individuals, 5% African American individuals, and 5% other underrepresented populations. The CCPM biobank is also significantly larger than the 1KGP, with 73,346 samples, over 20× more samples than the 1KGP.

Similar to our analysis for 1KGP populations, we evaluated GenoSiS cohorts at the population level using predicted genetic similarity groups to account for ambiguities in racial and ethnic categories or misclassification of race/ethnicity during clinical visits. Ancestry inference was based on projections of CCPM samples to ancestry clusters in the TPG and the Human Genome Diversity Project (HGP) (Fairley et al. 2020) reference panels. Samples are assigned a probability for each ancestry cluster and are then assigned the label of the cluster with the highest probability. CCPM adopts group labeling approaches that follow recommendations on population descriptors from the 2023 NASEM report (National Academies of Sciences, Engineering, and Medicine and Committee on the Use of Race, Ethnicity, and Ancestry as Population Descriptors in Genomics Research 2023) and are defined at the superpopulation level. For CCPM labeling information, see Supplemental Table S2. Given the size of the biobank, cohorts from other methods were not available for comparison.

**Figure 6.**
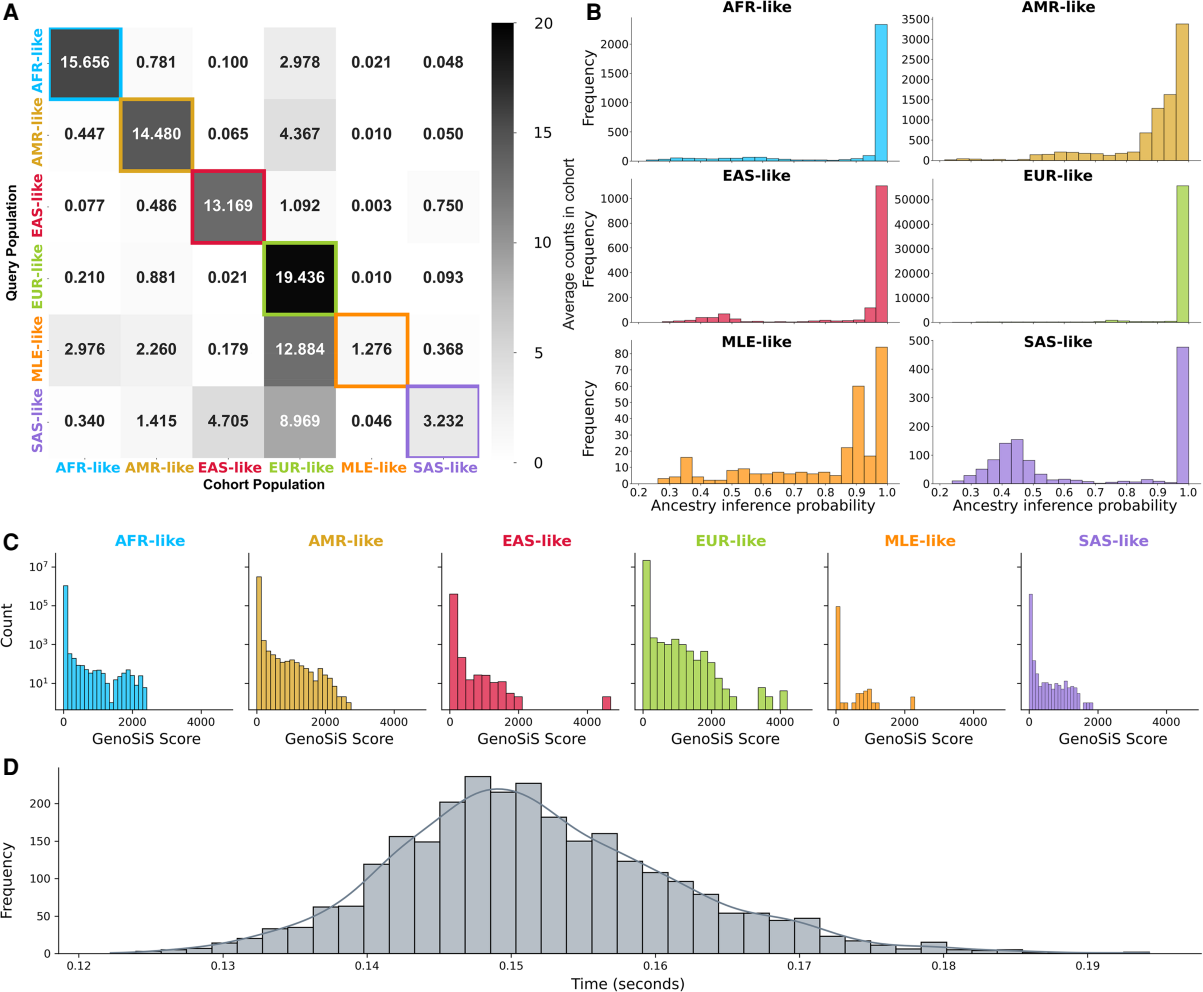
Average occurrence of each population for CCPM cohorts of size 20. (*A*) Average CCPM group counts for cohorts generated by GenoSiS plotted as a heatmap. Group labels for the query samples are listed on the *left* axis, and the labels of sample that appear in their GenoSiS cohort appear on the *bottom* axis. (*B*) CCPM ancestry inference probabilities for CCPM populations. (*C*) GenoSiS scores for CCPM groups. (*D*) GenoSiS search and aggregation times for CCPM's 73,346 biobank samples.

GenoSiS found cohorts predominantly from populations that matched the query samples’ population (Fig. 6A). The exceptions were from MLE- and SAS-like populations, in which the cohorts were most similar to the trained categories of EUR-like ancestry. The discrepancies with the MLE- and SAS-like populations are driven by two factors likely to affect many biobank-backed clinical analyses: small populations and ambiguous ancestry prediction. The MLE- and SAS-like populations were least represented in the CCPM biobank, constituting just 0.3% and 1.6% of the total, respectively. Variable sample sizes are a known source of bias for methods that rely on dimensionality reduction (Stockwell and Peterson 2002; Bean et al. 2012; Berahmand et al. 2024). Compounding the small populations was a shift in the quality of the ancestry inference for these two populations (Fig. 6B). The MLE- and SAS-like cohorts also had the lowest-scoring cohorts (Fig. 6C; Supplemental Fig. S3). The issues arising from small populations and ancestry inference highlight the importance of genetic-distance-based cohort creation, which GenoSiS is the only method designed to accomplish at scale.

### Query runtime

As the number of samples in biobanks continues to grow and the demand for patient-centered results increases, the speed at which we can find cohorts will become critical to their usefulness. GenoSiS maintains tractable runtimes on modern, large-scale data sets (e.g., more than 10^5^ of individuals, more than 10^10^ pairs). Although other measures of pairwise similarity may be faster and are optimized for initial readout, the network learned by GenoSiS enables rapid query of patient-matched cohorts, with subsequent queries after model training taking less than a second. This enables flexible analyses for the user, varying cohort size in an efficient manner. Among the 74,346 CCPM samples, GenoSiS can search for a single query in less than one-tenth of a second (∼0.025 msec) on a single c3-highmem-22 compute instance in Google Cloud. Because each query (one segment) can be performed independently (i.e., queries by segment do not depend on other segments), the runtime for a single sample (many segments) can be further improved using additional threads or specialized hardware accelerators. These results demonstrate that GenoSiS is fast enough to empower many patient-centric analyses, including point-of-care applications (Fig. 6D).

## Discussion

GenoSiS is a rapid, genome-wide search for genetically similar individuals that scales to large biobanks. GenoSiS is inspired by early genomic similarity detection methods while leveraging new technologies to rapidly match query samples to representative cohorts. Although existing methods (Nicholson et al. 2002; Purcell et al. 2007; McVean 2009; Manichaikul et al. 2010; Duforet-Frebourg et al. 2016) offer static clusters and N × N similarity metrics, using SVS eliminates the need to recompute significant portions of the matrix when adding new samples. With GenoSiS, we move toward inclusive research in precision medicine and motivate further research for query-based cohorts over finite ancestry labels. Rapid and accurate identification of these representative cohorts can be used to support real-time clinical studies and develop evidence-based precision treatment plans for physicians, researchers, and other members of clinical teams. Additionally, the identification of these cohorts could have promising implications in the exciting pursuit of inclusive genome wide association studies (GWAS), polygenic risk scores (PGSs), and other statistical analyses as biobank diversity continues to catch up with our increasingly diverse global human population (Gaynor et al. 2024).

A key risk to finding patient-matched cohorts is when insufficient patient diversity within an institution's biobank fails to create a large enough cohort (see “Cohorts with poor representation”). We can greatly reduce this risk by expanding the pool of potential patient matches via other institutions’ biobanks, so long as patient data remain private. One solution is a federated machine learning approach (Rieke et al. 2020), in which each institution trains a standardized learning model on its own local data and shares only the learned model parameter updates with a central server for aggregation, a strategy that precludes the need to share raw data. We believe that GenoSiS can easily accommodate this model by adopting a common training set curation process across collaborating institutions and by leveraging ideas from standard federated learning for handling heterogeneous data. In this setting, GenoSiS will need to work across private databases housing different subpopulations, some or all of which may not share statistical distributions with the queries. The recent introduction of a form of dimensionality reduction into SVS (Aguerrebere et al. 2023; Tepper et al. 2024) that improves search recall across differing subpopulations paves the way for GenoSiS to be applied in a federated setting.

### GenoSiS performance on deCODE closely related individuals

Treating deCODE's IBD method as a reliable measure of truth, Figure 2A supports the claim that relatedness for family members up to 6° is complicated. Although there is a strong signal for samples that are separated by one meiotic event (e.g., parent/child) and samples that are unrelated, the remaining signals (e.g., grandparent/child siblings, cousins, etc.) are less defined. GenoSiS is not an IBD method, but designing the search to perform at the 1 cM segment level allows for a reasonable comparison to these IBD results. Figure 2B demonstrates that GenoSiS scores have a similar distribution to IBD scores, with small differences. We note that GenoSiS scores for first-degree relatives have a wider distribution than IBD scores, and GenoSiS scores are slightly right-shifted. Though the distributions are mostly in agreement, the discrepancies are likely explained because IBD methods prefer to be careful when labeling IBD segments. Many IBD methods, for example, will only label regions IBD if the segment is ≥3 cM in length. The current aggregation and scoring method for GenoSiS is not designed to consider this level of granularity. Furthermore, when scores exceed the ∼50% shared centimorgans (e.g., pairs separated by two meiotic events), we note these samples are siblings and share genetic data on more than one haplotype.

### GenoSiS performance on 1KGP ancestry populations

Much like relatedness, uncovering population structure for human genetic data is a complex task. As discussed in our introduction, we elect to examine population structure from the lens of ancestry, in which ancestry labels are assigned within the 1KGP data. The assumption of truth for these analyses (Fig. 3) is that a cohort with more samples labeled with the same subpopulation as the query is more correct than a cohort with samples labeled with the same superpopulation as the query is more correct than a cohort with samples labeled outside of the query's superpopulation.

To evaluate GenoSiS cohort correctness against this standard, we benchmark against cohorts defined by three PLINK scores: (1) DST, (2) pi-hat, and (3) kinship. PLINK DST score is a rough approximation for pairwise IBS distance, in which IBS distance is a function of measuring the number of markers at IBS0 (e.g., AA, aa), IBS1 (e.g., AA, Aa), and IBS2 (Aa, Aa). DST values range from zero to one. A score of zero indicates no genetic relatedness, whereas a score of one indicates genetic clones. PLINK pi-hat score is a measure of proportion IBD. Likewise, a pi-hat score of zero indicates no genetic relatedness, whereas a score of one indicates genetic twins. Finally, the King-robust estimator performs relationship inference to estimate pairwise kinship coefficients. Here, duplicate samples have kinship scores of 0.5 (not one), first-degree relatives have scores of ∼0.25, second-degree relatives have scores of ∼0.125, and so on. Furthermore, negative kinship scores indicate samples from different populations. The distribution of GenoSiS and PLINK scores is shown in Supplemental Figure S2.

Figure 3 demonstrates cohort correctness at the subpopulation level. First, we note that for all four analyses, there is a strong diagonal signal for superpopulation labels (outlined in colored boxes), indicating that each method can at least identify cohorts that mostly match a query's superpopulation. Second, in all four analyses, there is a slightly weaker but still apparent diagonal signal for most subpopulations, indicating that these methods often identify cohorts that largely match a query's subpopulation. Evaluation at the subpopulation level demonstrates some unique patterns between these methods. For example, PLINK pi-hat (Supplemental Fig. S1B) shows a strong signal in which samples of GBR ancestry appear in cohorts for query samples that are not of GBR (or European) ancestry. This pattern is particularly noticeable for query samples of African ancestry. PLINK DST cohorts (Supplemental Fig. S1A) identify cohorts that are more consistent with the query subpopulation but still occasionally include samples outside of a query's superpopulation (e.g., African and American queries). King-robust coefficient and GenoSiS cohorts consistently generate the most correct cohorts at the super- and subpopulation level, with GenoSiS cohorts being slightly more in line with subpopulation labels compared with the King-robust coefficient.

Figure 3, B and C, supports results shown in Figure 3A with decaying values of *k*. All methods perform well for most superpopulations (gray lines), but the subpopulation level shows some performance differences. For each method, GenoSiS reports equal or better cohorts as *k*-values increase from one to 20.

### GenoSiS performance on CCPM data

Analysis on the CCPM biobank samples, shown in Figure 4, demonstrates a similar pattern to the analysis on the 1KGP samples. That is, for most ancestry groups, GenoSiS cohorts comprise other samples from the same ancestry as the query sample. With CCPM data, however, GenoSiS cohorts for samples of 1KGP + HGP-MLE-like and 1KGP + HGP-SAS-like ancestry comprise a higher proportion of 1KGP + HGP-EUR-like samples on average than would be expected. Supplemental Figure S3 further reports GenoSiS scores for the same set of data. This analysis shows that although there is an overrepresentation of European samples in queries for 1KGP + HGP-MLE-like and 1KGP + HGP-SAS-like samples, the GenoSiS scores for these 1KGP + HGP-EUR-like samples remain relatively low (i.e., not inflated over the GenoSiS scores for samples of the same ancestry group). Furthermore, some of the poorer performance of 1KGP + HGP-MLE-like and 1KGP + HGP-SAS-like populations could be owing to how population groups were defined in CCPM. During this process, every individual is assigned to a single group even if there is nearly the same amount of support for assignment to a different group (e.g., admixed samples), which could convolute some of these group assignments. Of the 73,346 samples in the CCPM biobank, ∼2% are 1KGP + HGP-EAS-like, 1.6% are 1KGP + HGP-SAS-like, 4.1% are 1KGP + HGP-AFR-like, 0.3% are 1KGP + HGP-MLE-like, 80% are 1KGP + HGP-EUR-like, and 12% are 1KGP + HGP-AMR-like. We note that the two ancestry groups with the poorest cohort quality (higher number of outpopulation samples in the cohort) also have the fewest number of samples represented in the database. It has been well established that an imbalanced set of classes (i.e., ancestry groups) complicates *k*-nearest neighbor (KNN) search algorithms (Fernández et al. 2017; Shi 2020). The consequence nor the solution for this imbalance is straightforward in this context, but we investigate the limitations of these imbalances and other possible biases and discuss them below.

### GenoSiS performance with different database–query representations

As shown in Figure 3, B and C, and discussed above, the quality of a GenoSiS cohort can depend on the composition of the database of samples. For example, if GenoSiS creates an index on a database of exclusively European individuals, the GenoSiS cohort for an African query sample will necessarily constitute only European individuals, likely with low GenoSiS scores. To further investigate the scores for GenoSiS cohorts generated in these scenarios, we created five isolated databases for each of the five superpopulations in the 1KGP data set and performed isolated queries for each pair of superpopulation groups. The results from these experiments are shown in Figure 5.

As expected, we first observe that for all query populations, the databases that return the highest GenoSiS scores are those that have the same population as the query. Further, we observe that the samples that contribute most to these higher scores come from samples within the same subpopulation as the query sample. A second observation is that for all queries, the African database returns higher GenoSiS scores for non-African queries than other databases do for outpopulation queries. Specifically, queries of American ancestry observe cohorts with higher GenoSiS scores for African databases than for other databases. This observation is consistent with what we know about 1KGP American samples having admixed ancestry and 1KGP African samples having a more diverse representation over other 1KGP populations (Gouveia et al. 2020; Micheletti et al. 2020). We include additional comments regarding the task of better understanding GenoSiS cohort quality for real-world databases (i.e., biobanks) and other comments on the current limitations of GenoSiS in our section on future work.

### Future work and limitations

#### Data preprocessing

Future versions of GenoSiS could benefit from analysis of optimal slice sizes (e.g., >1 cM, <1 cM, variable slice sizes, etc.).

#### Encoding vectors

GenoSiS generates positional encodings derived from the binary encodings; however, nominal encodings could be considered in place of binary encodings to account for the multiallelic nature of human variation.

#### Siamese training

Future work could focus on serving underrepresented groups by asserting a more robust training process. Some possibilities might be to include more admixed populations and high-variation samples, exploring the option to select a more representative chromosome or set of segments across many chromosomes, adding features of the data (e.g., multiallelic sites, structural variants, etc.), adding other tasks (e.g., maximize differences between ancestry groups), or training on different distance metrics, especially if nominal encoding vectors are added. Applying Summix (Arriaga-MacKenzie et al. 2021), a tool that provides ancestry adjusted allele frequencies, to training data would be an immediately useful step to address more than one of these ideas.

Furthermore, if other types of data are considered as input to the model (e.g., methylation, expression, health records, etc.), new distance metrics could be considered. The architecture of a Siamese training process allows for any distance metrics to be applied in the loss function, and downstream steps of GenoSiS are already designed to easily handle different kinds of embedding vectors. As such, the applications for new data and more complex training decisions are reasonable additions.

#### SVS index and search

More specific recommendations for choosing an appropriate *k* should be quantified. Currently, GenoSiS is set up to run with mostly default options, using the Vamana index with L_2_ distance. Future work should explore other indexing, searching, and loading options. Furthermore, advanced vector compression techniques could provide acceleration and memory footprint reductions.

#### Aggregation

During development, we investigated other methods for aggregation including merging gaps, setting a minimum limit to consecutive segment inclusion, and using the reported SVS score as a scoring technique. Popcount was most consistent with our initial evaluation criteria, but we assert that further exploration of these and other scoring algorithms (e.g., HMM) has an interesting path forward.

#### Controlled simulations of confounding variables

Although GenoSiS was evaluated across different sequencing platforms (SNP array with imputation, WGS) and populations (1KGP, Icelandic pedigrees), many variables that differ across data sets remain untested. Sequencing error, marker density variation, and admixture each have the potential to affect cohort quality in ways the current evaluations cannot isolate. Comprehensive simulations using software like msprime (Baumdicker et al. 2022) or SLiM (Messer 2013) would allow controlled testing of these variables. We plan such experiments, particularly for populations with complex admixture histories and institutions relying on array-based genotyping.

#### Index construction and query runtime comparisons

GenoSiS queries 73,346 samples in under one-tenth of a second per segment, but we do not benchmark runtime directly against PLINK or King-robust. These methods compute N × N pairwise matrices and are not designed for single-query use, making direct comparison difficult. Index construction time was not characterized here, as it is highly sensitive to GPU/CPU availability and configuration, which varied across our compute environments. A systematic runtime comparison against existing methods remains an important gap. With that said, the GenoSiS embedding model is quite small by modern AI standards (fewer than 1 million parameters) and can easily run inference using either GPU or CPU compute.

#### Generalization across sequencing modalities and populations

Embeddings trained on 1KGP WGS transferred successfully to CCPM targeted sequencing data, and cohort quality was consistent across most ancestry groups in both data sets. Two limitations apply. Performance degraded for MLE- and SAS-like populations in CCPM, driven by small sample counts and ambiguous ancestry assignments. African-ancestry individuals showed reduced embedding quality owing to reference bias in the positional encoding and the previously noted higher variant density. Together, these results suggest GenoSiS generalizes well across sequencing modalities but has limitations when the matching population cohort is small or when query and indexed populations have high variant density.

#### Potential applications

We are currently exploring using GenoSiS to improve risk prediction for widely used biomarkers like hemoglobin A1c (HbA1c) level, a standard metric for determining if a person is prediabetic or diabetic. Although HbA1c is useful, it has limitations as environmental factors and genetic variation affect an individual's level. Our research aims to test if cohorts generated by GenoSiS can provide better background distributions for A1c interpretation, accounting for these genetic influences to refine risk assessment and lead to more personalized health insights.

## Methods

### Haplotype embedding model training

#### Model architecture

The haplotype embedding model takes positional haplotype encoding vectors as input (see Methods section “GenoSiS architecture,” subsection “Data preprocessing”) and outputs a 512-dimensional embedding vector. The model consists of a 1D convolutional neural network (CNN) with six blocks followed by a global mean pooling operation and a fully connected layer to output the embedding vector. Each convolutional block consists of a 1D convolution followed by dropout, batch normalization, *gelu* activation function, and max pooling. Each convolutional block has a kernel size of six and stride of one. The number of input and output channels is defined by *C*_in_ = 32*i*, *C*_out_ = 32(*i* + 1); *i* = [1..6), where *i* denotes the *i*th convolutional block. For the zero block, we have *C*_in_ = 1, *C*_out_ = 32. Max pooling kernel size is three, and the output embedding dimension is 512.

#### Siamese training

The GenoSiS embedding model's training objective was to learn mapping from genomic segments to embedding vectors such that Euclidean distances in embedding space reflect underlying genetic similarity. To accomplish this, we trained the model using a Siamese learning objective in which we minimize the difference between embedding distance and genotype distance. During training, pairs of segment-matched positional haplotype encoding vectors were presented to two CNNs with shared weights. The training objective was to minimize the mean squared error between two output embeddings’ cosine similarity and the ground-truth precomputed cosine similarity between their corresponding raw haplotype encoding vectors (see Methods section “GenoSiS architecture,” subsection “Data preprocessing”). Gradients along both CNN paths were mean aggregated and then used to update weights. The model was trained for 200 epochs with a batch size of 1024. For the optimizer, we used AdamW (Zhou et al. 2024) with a *base learning rate* = 10^−3^, β_1_ = 0.9 and β_2_ = 0.999, and a weight decay = 10^−4^. The base learning rate was scaled using torch's CosineAneallingWarmRestarts learning rate schedule with the parameters T_0 = 17328, T_mult = 1, and eta_min = 1 × 10^-6^. Training and validation loss curves can be found in Supplemental Note 3. A visualization of embedding quality at different stages of training can be found in Supplemental Note 4.

#### Training set

To train our model, we used positional encoding vectors (see Methods section “GenoSiS architecture,” subsection “Data preprocessing”) from the 1KGP data, Chromosome 8 (169 segments, 3.8 million variants). As we are only interested in quantifying similarity between the same segments, we only train on pairs within the same segment. In this system, we choose cosine similarity as our ground truth and specify our output embedding vectors to be of length 512. For the split of segments used for training, testing, and validation sets, see Supplemental Table S3.

To obtain pairs of training samples that span a wide, uniform range of similarities, we do the following:

for each sample S in the 1KGP:
 for each subpopulation P in the 1KGP subpopulations:
  randomly select 5 samples from P.
  pair each random sample with S.
  compute pairwise similarities for each pair.
divide pairs by similarity values into 20 bins.
randomly select at most 500 pairs from each bin.

We then split the 169 segments into train (80%), test (10%), and validation (10%) sets to ensure the model validates/tests on unseen genomic regions.

### GenoSiS architecture

The GenoSiS workflow consists of four major steps (Fig. 1): (1) preprocessing data, in which input VCF files are split into one centimorgan encoding segments; (2) generating embedding vectors from segment encodings with a trained neural network; (3) building ANN search indices from embedding vectors; and (4) searching the indices to report the final cohort. A general overview can be seen in Figure 1, and further details for each step are described below.

#### Data preprocessing

##### Input and intermediate files

Genotype data should be formatted as a phased *VCF file*, compressed, and indexed with bgzip and tabix.

*Map files* can be obtained from various sources and serve as a reference for the interpolated map generated in an intermediate preprocessing step. We use the build GRCh38 PLINK-format genetic map out of Washington University; links are provided below and in our GitHub repository.

To appropriately estimate start and end base-pair positions for the next step, we map an appropriate positional centimorgan to each base-pair coordinate that appears in the input VCF file. An i*ntermediate map file is generated by interpolating positional centimorgan data* for each base pair position witnessed in the VCF using the centimorgan data provided in the reference map file.

After the interpolated map file is created, the start and end points for all 1 cM segments are recorded in a *segment boundaries file*.

##### Slice VCF

For most of the remaining steps (excluding the final aggregation step), we process 1 cM segments independently. We choose a length of 1 cM to align with the IBD-method consensus of minimum length for IBD (2–3 cM). In this step, the input VCF file is parsed into 1 cM segments starting and ending at the segment boundaries identified in the previous step. All output segments from this step are written to independent VCF files, including the original header. Once VCF slicing is complete, each procedure can be completed independently for each segment.

##### Binary haplotype encoding vectors

To generate binary haplotype encoding vectors, GenoSiS encodes nonreference and reference alleles as one and zero, respectively. GenoSiS encodes biallelic variants separately, creating two binary haplotype encoding vectors for each sample (one vector for each of two genetic parents, or haplotypes). First, we record all genotypes in a data structure we call “variant major format (VMF),” in which rows are variants and columns are samples (as formatted in a VCF file). From VMF, we generate transpose to sample major format (SMF), in which rows are samples and columns are variants.

##### Positional haplotype encoding vectors

Binary haplotype encoding vectors are extremely sparse. From these sparse encodings, we create dense, positional haplotype encoding vectors. Here, we encode the centimorgan position (from the interpolated map file) only at sites marked as nonreference. By encoding the centimorgan position of a nonreference allele, we preserve the location of variants along a segment.

#### Generate embedding vectors

With our trained model (see first Methods section “Haplotype embedding model training”), we generate *haplotype segment embedding vectors* for all segments in all chromosomes by running inference on the model using the *haplotype positional encoding vectors* as input.

#### Creating index

For each 1 cM segment's embedding vectors, we build an ANN *search index using default settings from the Intel SVS*. Although SVS is one of the highest performing vector search databases, there are other solutions (Douze et al. 2026) that could be used in its place.

#### Searching index

A *SVS search* takes haplotype segment embedding vectors and SVS segment indices as input and returns the KNN for each query sample at each segment. Here, the selection of *k* is a tunable parameter, and we consider the significance and interpretation of different *k*-values in our discussion section.

#### Aggregate segments

To report a genome-wide representative cohort, we aggregate SVS search results from each segment's index into a binary vector denoting presence or absence in top *k*’s for all segments in a single chromosome. Samples with high similarity to the query will be dense, and those with low similarity will be sparse. To report a final cohort, we perform a summation operation for all match vectors. For aggregation at the chromosome level, we add up sample scores for each chromosome and report a single score for all match-sample IDs that appeared one or more times in the top *k*’s across all segments. The final cohort considers all match scores from each chromosome, ranks them by order of decreasing values, and reports top *k* samples genome-wide.

### Experiments

#### Closely related individuals

Tracking relatedness among family members by number meiosis is complicated because higher degrees of relatedness include various relationships with different inheritance patterns. For example, second-degree includes both grandparent/grandchild and full siblings. Full siblings share genetic data on two haplotypes whereas a grandparent/grandchild pair shares genetic data only on one haplotype. This arrangement leads to varying amounts of shared DNA beyond straight forward percentage expectations. This pattern continues with increased degrees of relatedness to include more and more relatives.

Using six families from deCODE's database and 608 trio samples from the 1KGP, we evaluate the performance of GenoSiS on closely related individuals. For the deCODE analysis, we create a GenoSiS index for the complete set of all families and query all samples to generate their GenoSiS cohorts. Similarly, for the 1KGP trio analysis, we created a single index for all 608 samples and queried all samples for their GenoSiS cohort. We label pairs of individuals by the number of meiotic events (i.e., generations) by which they are separated, and we add a label “unrelated” for individuals who come from separate families. We benchmarked the correctness of deCODE GenoSiS cohorts against an IBD tool provided by deCODE. This tool, like other IBD methods, computes pairwise IBD for all samples to quantify the length of shared segments of DNA between two samples. deCODE's IBD method, like most IBD methods, does not report a genome-wide score; instead, it reports pairwise IBD segments. The score reported in Figure 2A is a summation of IBD segments across both haplotypes.

#### Distantly related individuals

Using the 1KGP data with labels for superpopulation (e.g., AFR, EUR, etc.) and subpopulation (e.g., ESN, GBR) ancestry groups, we further investigate GenoSiS at the population level. We create a GenoSiS index for the complete 1KGP data set and query all samples to generate their GenoSiS cohorts. For each query, we record the subpopulation label of the query,and of the *k* cohort members (e.g., a query sample who is SAS, Gujarati [GIH] might have a cohort with 15 SAS–GIH, three SAS–BEB, one SAS–ITU, and one EUR–TSI). In Figure 3A, we demonstrate the decay of GenoSiS cohorts as more samples are included in the cohort (i.e., *k* increases from one to 20) and compare to cohorts inferred by other methods. We also report the average counts for all queries’ cohort populations and plot a heatmap of these averages in Figure 3, B and C. We did the same for PLINK DST, pi-hat, and kinship, and we plot their results in Supplemental Figure S1. As discussed, none of the PLINK tools report a representative cohort (i.e., KNN) in the same way GenoSiS does. To generate PLINK cohorts, we added a post hoc ranking step that selects the top *k* = 20 samples with the best PLINK scores (i.e., most similar samples) for each query sample.

#### F_*ST*_

*F*_ST_ values for 1KGP subpopulation pairs were obtained from Privé et al. (2022). In Figure 3D, the 1KGP subpopulations are grouped on each axis such that they are adjacent to their respective superpopulations, and then, we generate a simple heatmap of *F*_ST_(*x*, *y*) for all combinations of subpopulations (*x*, *y*). In Figure 3E, for each 1KGP subpopulation, we use GenoSiS and other tested methods to aggregate cohorts using previously described methods and count the number of results from each subpopulation. We then plot the percentage of samples in each cohort from subpopulations with an *F*_ST_ value less than or equal to *x*, where *x* ∈ [0, 0.15] to generate an empirical density function for each method.

#### CCPM

We repeat the same process we used to generate Figure 3, B and C, to generate Figure 4, A and B, using more than 73,000 samples from the CCPM biobank. As there are no subpopulations associated with this data set, we label only CCPM group labels. We compute *F*_ST_ values for all 22 autosomes from CCPM data with PLINK2's ‐‐fst option.

#### Quality

To investigate how GenoSiS performs on different database/query combinations, we generated five separate databases for each of the five 1KGP superpopulations (AFR, AMR, EAS, EUR, SAS). We then performed queries for all samples in all combinations of database/query combinations (e.g., all AFR sample query into an AFR database, all AFR samples query into an EAS database, etc.). In these experiments, there are 895 African samples, 491 American samples, 586 East Asian samples, 634 European samples, and 602 South Asian samples. Figure 5 shows the results of these queries as a histogram of GenoSiS scores.

#### Timing

To evaluate the speed of GenoSiS, we report timing for our largest available data set (CCPM biobank data). In compliance with CCPM data sharing agreement, we perform our search on a c3-highmem-22 compute instance in Google Cloud (176 GB, Intel Xeon platinum 8481C 2.70 GHz) provided to us by our health data compass support team. We report search times for a single query, in which each sample constitutes two queries (i.e., two haplotypes), over all 2737 segments from the CCPM data. Each segment's search time was reported individually, and the distribution of these times are shown in Figure 4D.

### Data

#### 1KGP data

We use phased VCFs for 22 autosomes from 1KGP 30× on GRCh38; 3201 samples are labeled as five “superpopulations” (AFR, AMR, EAS, EUR, and SAS) and 26 as “subpopulations.” One sample with AFR–EUR descent is omitted from analyses.

#### deCODE families

From the deCODE database, we isolated Chromosome 18 for 10 families, each of which had at least four and up to six generations, totaling 274 samples. Siblings that were not full-siblings were pruned from the data set.

#### Colorado Center for Personalized Medicine

We use phased VCFs for 22 autosomes from the CCPM database. Of the 73,346 samples, roughly 23,000 were genotyped with the multiethnic genotyping array, whereas the remaining approximately 50,000 had exome sequences with a genomic backbone. All samples were imputed with TOPMed version 2. Ancestry labels are as follows: 1445 1KGP + HGP-EAS-like, 1201 1KGP + HGP-SAS-like, 3041 1KGP + HGP-AFR-like, 254 1KGP + HGP-MLE-like, 58,708 1KGP + HGP-EUR-like, and 8697 1KGP + HGP-AMR-like.

#### Map files

Map files are at https://bochet.gcc.biostat.washington.edu/beagle/genetic_maps/.

### Code availability

The code for running GenoSiS and the code used to generate figures and other data can be found at GitHub (https://github.com/kristen-schneider/genosis and https://github.com/ryanlayerlab/genosis_analysis, respectively). We have also created a snapshot of these repositories and provided them as Supplemental Code. Intel's SVS software can be found at GitHub (https://github.com/intel/ScalableVectorSearch).

## Supplemental Material

Supplement 1

Supplement 2

Supplement 3

Supplement 4

Supplement 5

Supplement 6

Supplement 7

Supplement 8

Supplement 9

Supplement 10

Supplement 11
