# Supplementary material for: Biobank-scale genotype similarity search and dynamic patient-matched cohort creation with GenoSiS
Source: Genome Res. 2026 Aug;36(8):1624–36. doi: 10.1101/gr.280278.124 (PMC13431173; doi:10.1101/gr.280278.124)
Supplement: Supplement 1 [file Supplemental_Fig_S1.pdf]

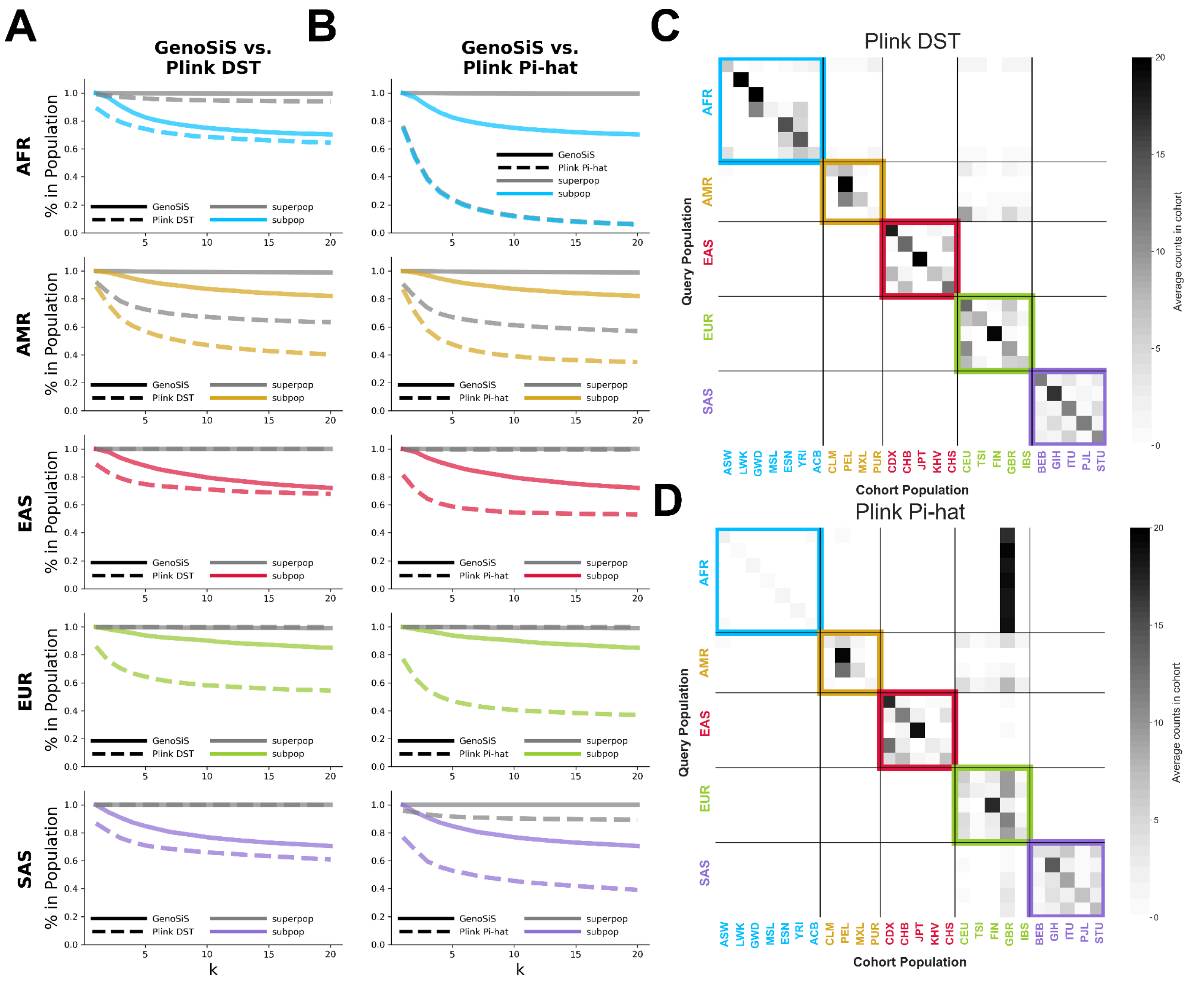

Figure S1. Super- and subpopulation percentages for TGP cohorts. AB The percentage of samples from the cohort which are in the super and subpopulation of the query samples as the size of the cohorts increase from 1 to 20. GenoSiS is plotted as a solid line while PLINK DST **(A)** and PLINK Pi-hat **(B)** is plotted as a dashed line. Lines for super populations are plotted in gray, while lines for subpopulation are plotted in a color corresponding to the population. **C** PLINK DSK and **D** PLINK Pi-hat. For both **C** and **D** super population labels for query samples are listed on the vertical axis and are colored accordingly. Subpopulation labels for samples in the representative cohort are listed on the horizontal axis and are colored according to their respective super population.
