# Supplementary material for: Biobank-scale genotype similarity search and dynamic patient-matched cohort creation with GenoSiS
Source: Genome Res. 2026 Aug;36(8):1624–36. doi: 10.1101/gr.280278.124 (PMC13431173; doi:10.1101/gr.280278.124)
Supplement: Supplement 2 [file Supplemental_Fig_S2.pdf]

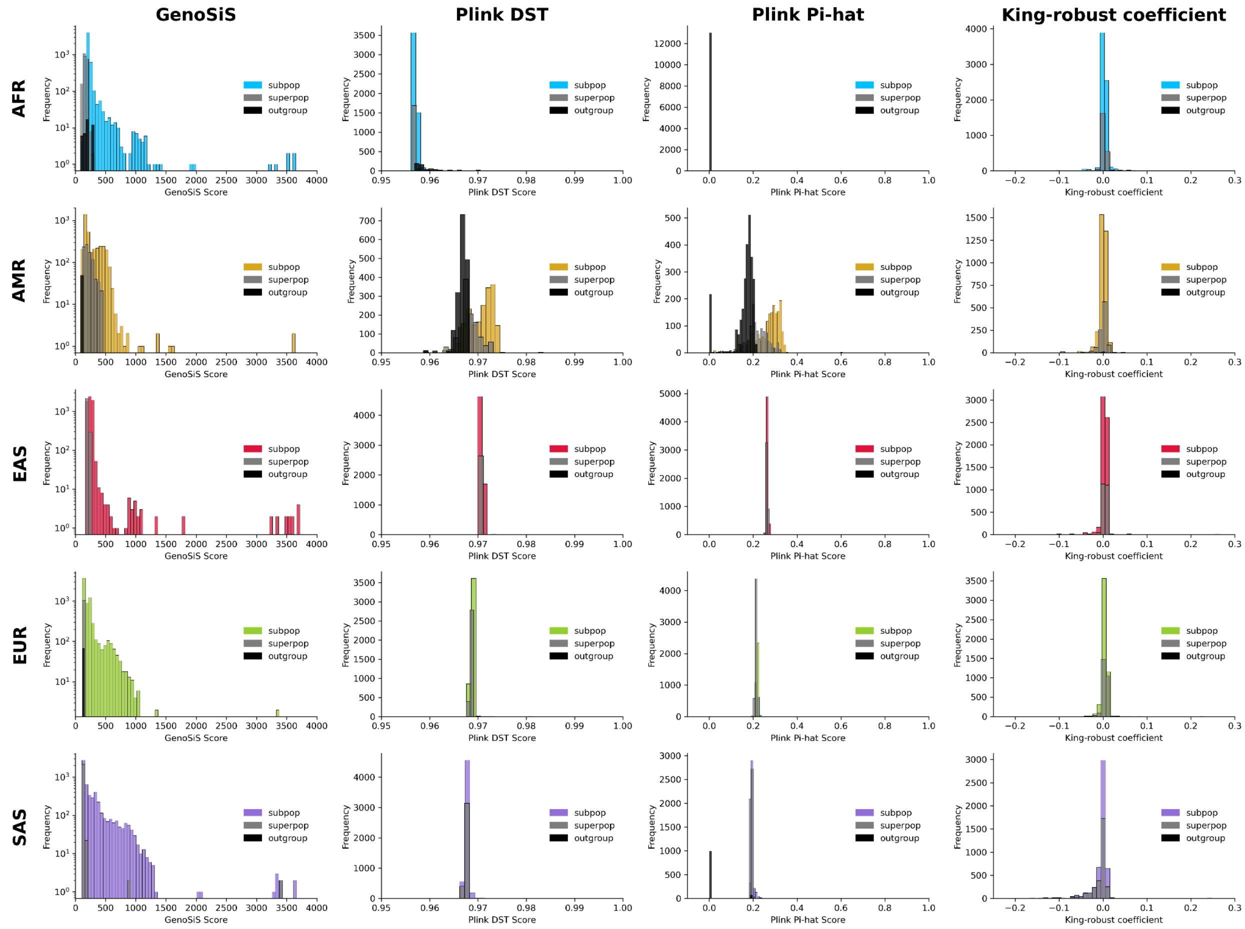

Figure S2. TGP cohort scores by population. Histograms of GenoSiS, PLINK DST, PLINK pi-hat, and King robust coefficient cohort scores for TGP data for *k*=20. TGP super populations are organized by row. Samples in the cohort which appear in the same subpopulation as the query sample are colored in yellow, red, green, blue, and purple for AFR, AMR, EAS, and SAS query samples accordingly. Samples in the cohort which appear in the same super population as the query sample are colored in gray. Samples in the cohort which appear outside of the query's super population are colored in black.
