# Supplementary material for: Biobank-scale genotype similarity search and dynamic patient-matched cohort creation with GenoSiS
Source: Genome Res. 2026 Aug;36(8):1624–36. doi: 10.1101/gr.280278.124 (PMC13431173; doi:10.1101/gr.280278.124)
Supplement: Supplement 3 [file Supplemental_Fig_S3.pdf]

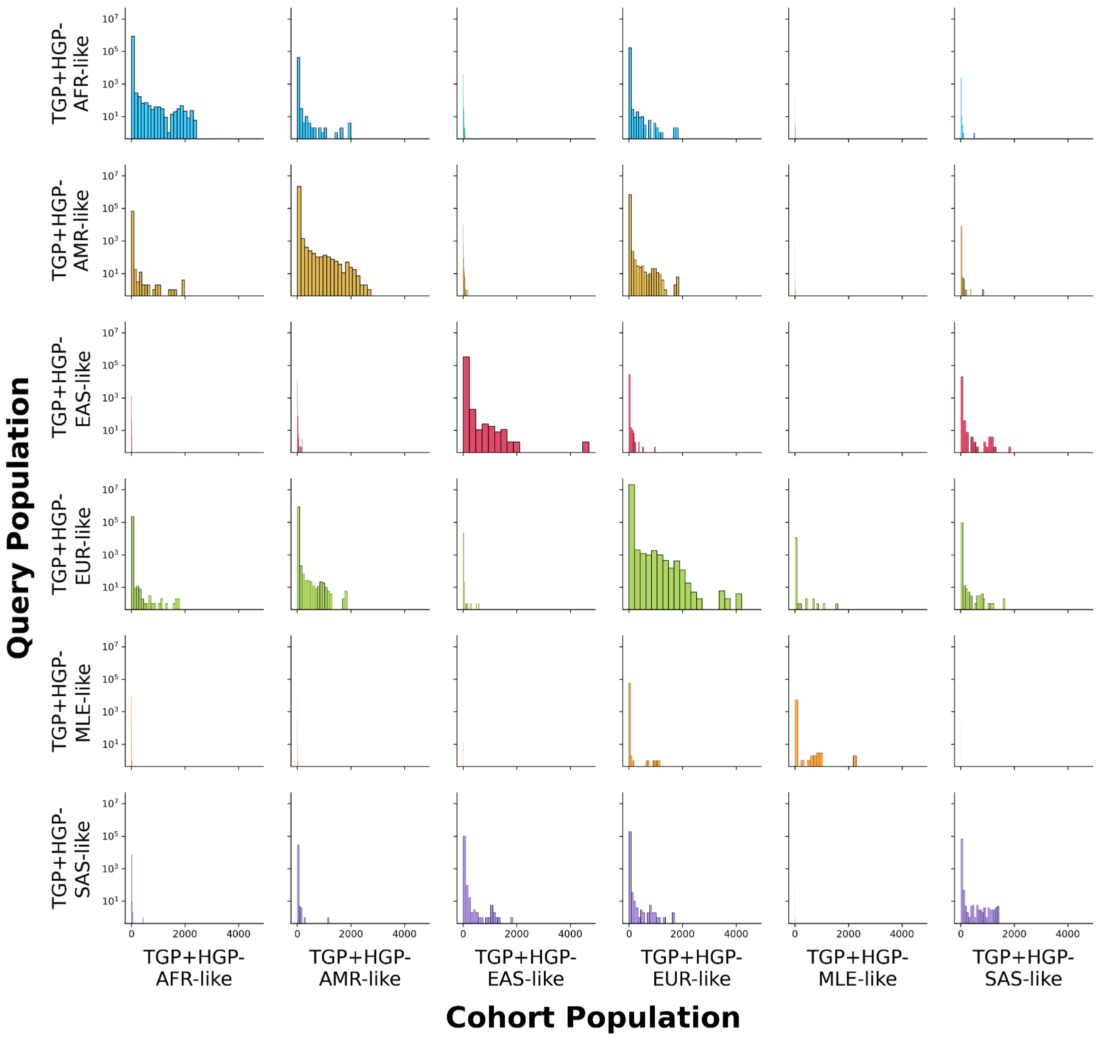

Figure S3. CCPM Biobank cohort scores by population. Histograms of GenoSiS cohort scores when *k*=20 for CCPM Biobank data. Query and cohort population labels are plotted in the same order for both axes.
