# Supplementary material for: Biobank-scale genotype similarity search and dynamic patient-matched cohort creation with GenoSiS
Source: Genome Res. 2026 Aug;36(8):1624–36. doi: 10.1101/gr.280278.124 (PMC13431173; doi:10.1101/gr.280278.124)
Supplement: Supplement 4 [file Supplemental_Note_1.pdf]

Supplemental Note 1: Divergence Between GenoSiS and Kinship-Based Similarity Metrics

To better understand the differences between GenoSiS and the kinship similarity metrics, we examined the case where the divergence between the two methods was most pronounced in Figures 3B and 3C. The search results for GBR (British) individuals showed the largest classification differences between methods.

GenoSiS consistently retrieved cohorts predominantly composed of GBR individuals, while the kinship-based approach tended to return CEU (Utah residents with Northern and Western European ancestry) individuals instead. The cohorts identified by GenoSiS were composed of approximately 60% GBR individuals, whereas the kinship-based cohorts contained roughly 60% CEU individuals (**Supp. Note Fig. 1.1**).

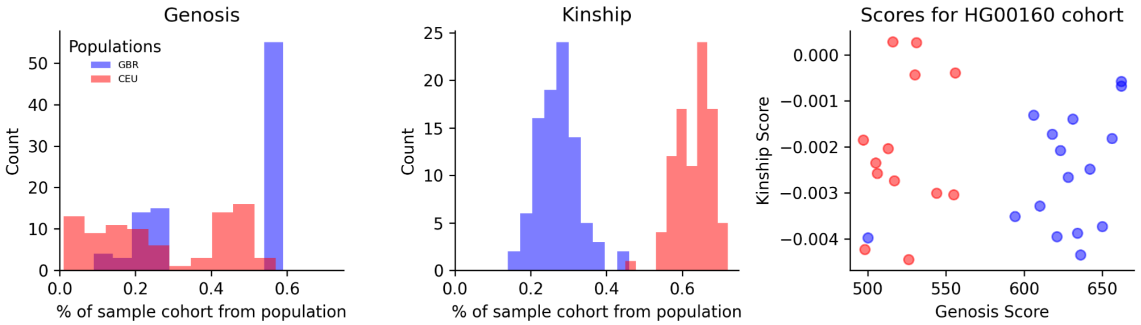

Supplementary Note Figure 1.1. The ancestry composition of cohorts constructed for GBR individuals using GenoSiS and the Kinship genetic distance metric, and a direct comparison of GenoSiS and Kinship scores for one GBR individuals's (HG00160) cohort.

Focusing on the cohort retrieved for GBR sample HG00160, the GenoSiS scores clearly distinguished between subpopulations, whereas the kinship scores did not. This discrepancy stems from the foundational differences between the methods. The kinship metric is based on the number of sites where two samples share no alleles (IBS0). While IBS0 is informative for identifying close relatives who typically have few or no IBS0 sites, it becomes less reliable for distantly related individuals, as IBS0 values converge toward population-level averages. In contrast, GenoSiS is based on Euclidean distance in genotype space, which captures more subtle patterns of variation and remains informative across the full spectrum of genetic relatedness. This enables more accurate similarity searches even among unrelated individuals.

This distinction is apparent in the top match comparisons for HG00160. According to the kinship method, the most related sample was the CEU sample NA12801, with 439,734 IBS0 sites shared with HG00160. GenoSiS ranked the GBR sample HG00137 as the closest match, which had 452,619 IBS0 sites. By the IBS0 alone, that NA12801 is more closely related. However, when incorporating other IBS metrics, the picture changes. HG00137 had 901,392 IBS1 sites with HG00160, compared to 897,862 for NA12801, indicating that HG00137 is more closely related in terms of IBS1. For IBS2, the difference was minimal: HG00137 shared 864,931 IBS2 sites with HG00160, while NA12801 shared 867,336, a difference of just 0.2%. These results reinforce our earlier point that IBS-based metrics, while effective for identifying close relatives, are less suited for detecting subtle population structure or for similarity search among distantly related individuals. GenoSiS, by leveraging genotype-space embeddings and Euclidean distances, provides a more robust and generalizable approach.
