# Supplementary material for: Biobank-scale genotype similarity search and dynamic patient-matched cohort creation with GenoSiS
Source: Genome Res. 2026 Aug;36(8):1624–36. doi: 10.1101/gr.280278.124 (PMC13431173; doi:10.1101/gr.280278.124)
Supplement: Supplement 5 [file Supplemental_Note_2.pdf]

Supplemental Note 2: Impact of Population Representation on Embedding Performance

Our analysis of the R^2 decline was intended to add transparency about potential inequities in model performance; however, we do not interpret this effect as indicating a population-specific performance issue. The R^2 decline between genotype distances and embedding distances was most pronounced in the AFR (African) samples but the correlation between genotype and embedding distance declines consistently with increasing segment density across all populations. The apparent concentration of the effect in the AFR population reflects the fact that AFR samples have a higher proportion of high-density segments. In all cases, the R^2 value is driven by the large number of sample pairs that are highly dissimilar. Since GenoSiS is designed for approximate *k*-nearest neighbor search, its performance is most critical among sample pairs with moderate to high similarity with the vast majority of matched cohort distances falling below a 1.0 (**Supp. Note Fig. 2.1**), indicating that the model performs is unlikely to be affected by the embedding correlation degradation.

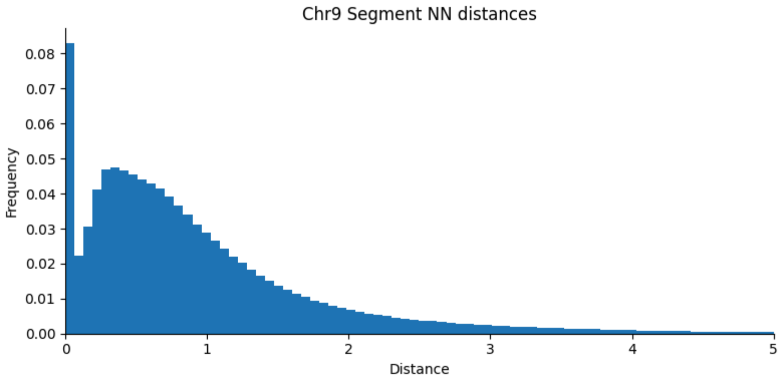

Supplemental Note Figure 2.1. The distribution of sample pair distances for chromosome 9 segments across all samples in matched cohorts.

To further investigate the effect of population representation, we trained a model with no AFR samples. Interestingly, in this model R^2 declines for all populations (**Supp Note Figs 2.2 and 2.3**). We reasoned that this model-wide degradation was due to both the large number of AFR individuals (nearly 30% of the training set) and that AFR samples contributed crucial training examples at both ends of the similarity spectrum (**Supp Note Fig. 2.4**). Their removal reduced the model's ability to generalize across the full range of genetic similarity. These results highlight the broader importance of diversity in training data. Inclusion of populations like AFR enhances overall model robustness.

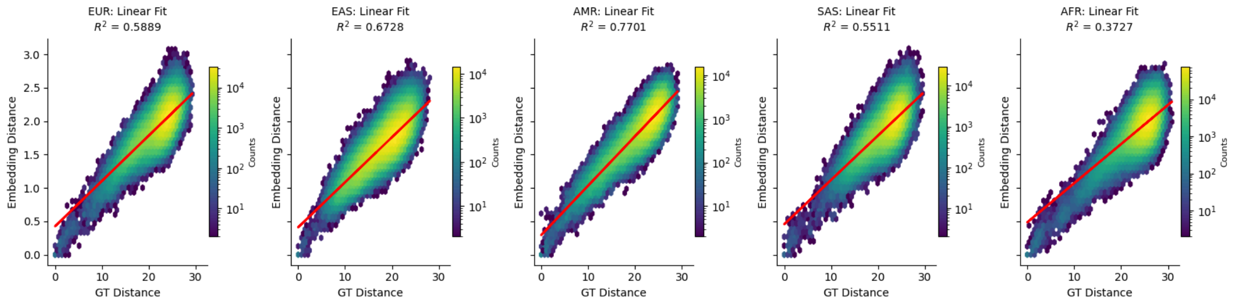

Supplemental Note Figure 2.2. The relationships between genotype distances and embedding distances among different populations using the when trained on all 1000 Genomes Project.

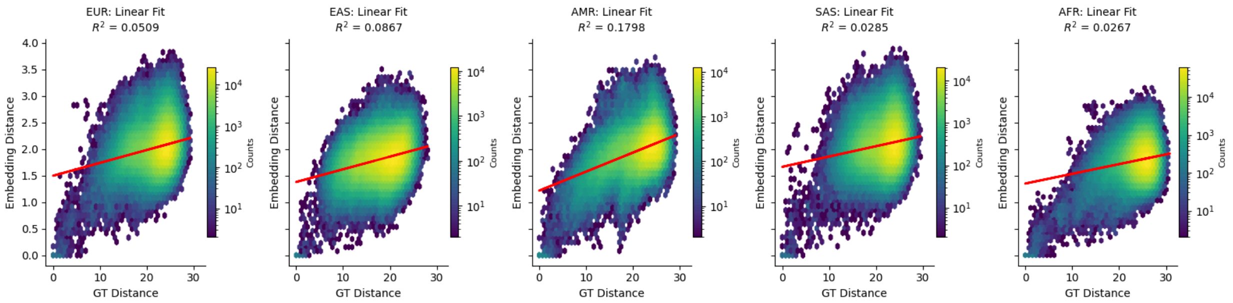

Supplemental Note Figure 2.3. The relationships between genotype distances and embedding distances among different populations when AFR samples are omitted from the training set.

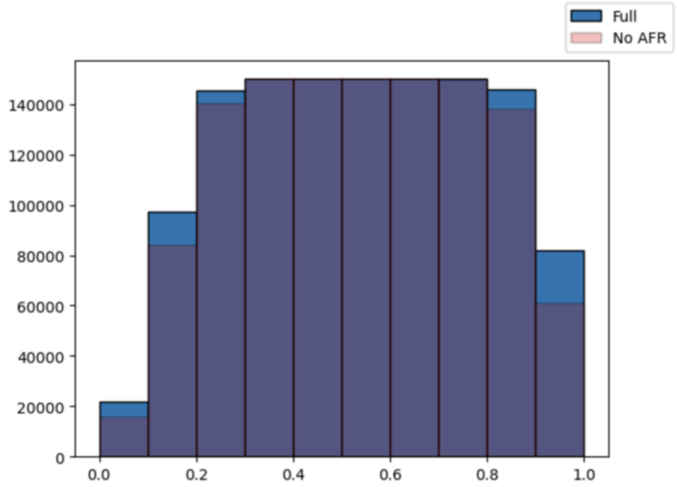

Supplemental Note Figure 2.4. Pairwise cosine similarity histograms for Full sample set and No AFR sample set training sets. Both sets were sampled in the same manner as outlined in the methods section
