# Supplementary material for: Biobank-scale genotype similarity search and dynamic patient-matched cohort creation with GenoSiS
Source: Genome Res. 2026 Aug;36(8):1624–36. doi: 10.1101/gr.280278.124 (PMC13431173; doi:10.1101/gr.280278.124)
Supplement: Supplement 6 [file Supplemental_Note_3.pdf]

Supplemental Note 3: Training and Validation Loss Curves

The training and validation loss and MSE curves across 200 training epochs (**Supp. Note Fig. 3.1**) provide insight into model convergence, training stability, and potential overfitting. Validation loss was computed at the end of each epoch. While the validation loss exhibits some epoch-to-epoch variance, likely due to the relatively small number of validation segments compared to the training set, we observe a consistent downward trend, indicating generalization improvements over time. Due to computational constraints, we did not perform extensive hyperparameter tuning or extend training beyond 200 epochs. However, based on the trend and our exploratory experiments, we believe any additional gains from further training would be modest.

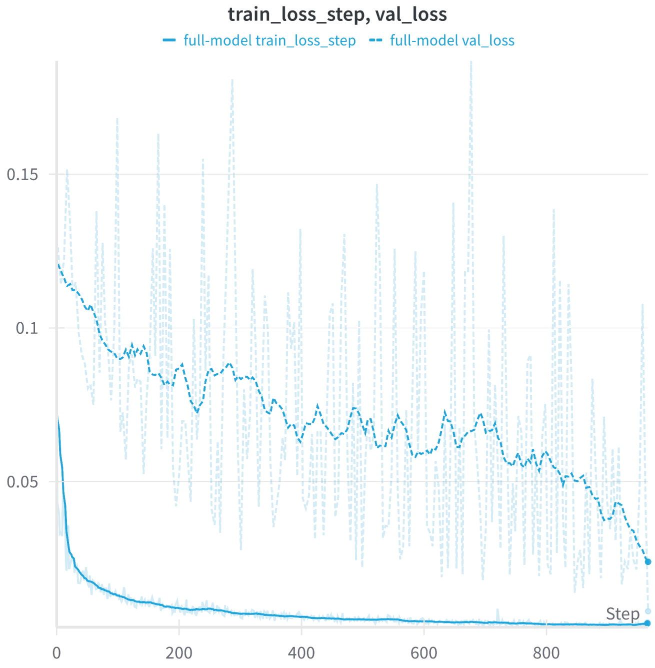

Supplemental Note Figure 3.1. Training and validation loss (MSE) curves for GenoSiS model across 200 epochs. Dark coloring indicates a running average of 20 previous points, and light coloring indicates original values.
