# Supplementary material for: Biobank-scale genotype similarity search and dynamic patient-matched cohort creation with GenoSiS
Source: Genome Res. 2026 Aug;36(8):1624–36. doi: 10.1101/gr.280278.124 (PMC13431173; doi:10.1101/gr.280278.124)
Supplement: Supplement 7 [file Supplemental_Note_4.pdf]

Supplemental Note 4: Embedding Quality Across Training

To visualize how embeddings improve with training, we tracked the change in R^2 values over time for a representative segment. **Supplemental Note Figure 4.1** shows hex bin plots comparing genotype distances (x-axis) and embedding distances (y-axis) for three models: (1) a randomly initialized model, (2) a model trained for 25 epochs, and (3) the fully trained model. Color intensity reflects sample pair density in each bin. As training progressed, the correlation between genotype and embedding distances tightened, demonstrating that the model was learning to encode meaningful genetic structure.

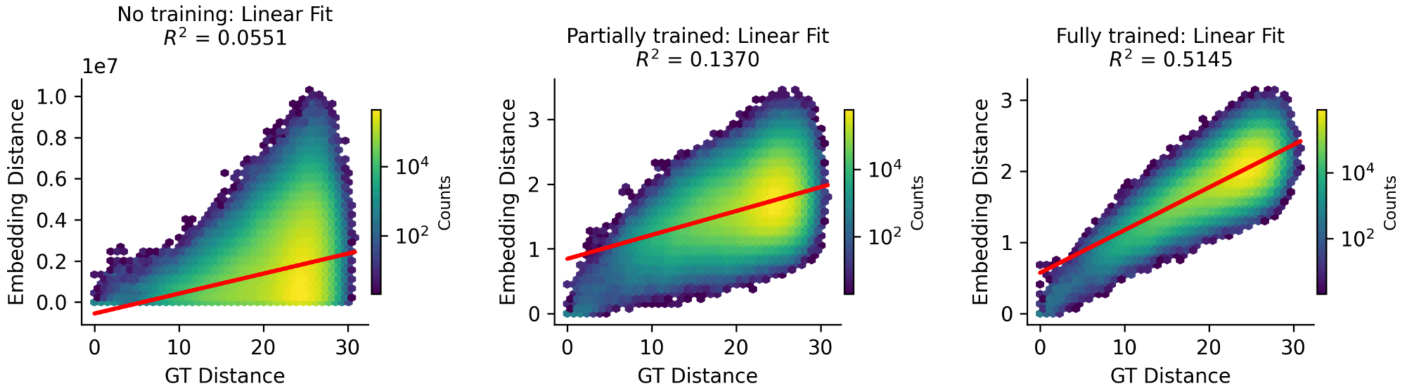

Supplemental Note Figure 4.1. The relationships between genotype distances and embedding distances at different stages of training for all 1000 Genomes Project sample pairs in chromosome 9, segment 75.
