# Supplementary material for: Biobank-scale genotype similarity search and dynamic patient-matched cohort creation with GenoSiS
Source: Genome Res. 2026 Aug;36(8):1624–36. doi: 10.1101/gr.280278.124 (PMC13431173; doi:10.1101/gr.280278.124)
Supplement: Supplement 8 [file Supplemental_Table_S1.pdf]

Supplemental Table S1. Thousand Genomes Project (TGP) Subpopulation and super population labeling.

Super Population	Subpopulation Abbreviation	Subpopulation Name
African (AFR)	ACB	African Caribbean in Barbados
African (AFR)	ASW	Americans of African Ancestry in SW USA
African (AFR)	ESN	Esan in Nigeria
African (AFR)	GWD	Gambian in Western Divisions in the Gambia
African (AFR)	LWK	Luhya in Webuye, Kenya
African (AFR)	MSL	Mende in Sierra Leone
African (AFR)	YRI	Yoruba in Ibadan, Nigeria
American (AMR)	CLM	Colombians from Medellin, Colombia
American (AMR)	MXL	Mexican Ancestry from Los Angeles, USA
American (AMR)	PEL	Peruvians from Lima, Peru
American (AMR)	PUR	Puerto Ricans from Puerto Rico
East Asian (EAS)	CDX	Chinese Dai in Xishuangbanna, China
East Asian (EAS)	CHB	Han Chinese in Beijing, China
East Asian (EAS)	CHS	Southern Han Chinese
East Asian (EAS)	JPT	Japanese in Tokyo, Japan
East Asian (EAS)	KHV	Kinh in Ho Chi Minh City, Vietnam
European (EUR)	CEU	Utah Residents (CEPH) with Northern and Western European Ancestry
European (EUR)	FIN	Finnish in Finland
European (EUR)	GBR	British in England and Scotland
European (EUR)	IBS	Iberian Population in Spain
European (EUR)	TSI	Toscani in Italy
South Asian (SAS)	BEB	Bengali in Bangladesh
South Asian (SAS)	GIH	Gujarati Indian in Houston, TX
South Asian (SAS)	ITU	Indian Telugu in the UK
South Asian (SAS)	PJL	Punjabi in Lahore, Pakistan
South Asian (SAS)	STU	Sri Lankan Tamil in the UK
