# Supplementary material for: Biobank-scale genotype similarity search and dynamic patient-matched cohort creation with GenoSiS
Source: Genome Res. 2026 Aug;36(8):1624–36. doi: 10.1101/gr.280278.124 (PMC13431173; doi:10.1101/gr.280278.124)
Supplement: Supplement 9 [file Supplemental_Table_S2.pdf]

Supplemental Table S2. Colorado Center for Personalized Medicine (CCPM) ancestry group-labeling.

**Abbreviation**	**Full CCPM labeling**
TGP+HGP-AFR-like	Thousand Genomes Project + Human Genomes Project-African-like
TGP+HGP-AMR-like	Thousand Genomes Project + Human Genomes Project-American-like
TGP+HGP-EAS-like	Thousand Genomes Project + Human Genomes Project-East Asian-like
TGP+HGP-EUR-like	Thousand Genomes Project + Human Genomes Project-European-like
TGP+HGP-MLE-like	Thousand Genomes Project + Human Genomes Project-Middle East-like
TGP+HGP-SAS-like	Thousand Genomes Project + Human Genomes Project-Central South Asian-like
