# Supplementary material for: Biobank-scale genotype similarity search and dynamic patient-matched cohort creation with GenoSiS
Source: Genome Res. 2026 Aug;36(8):1624–36. doi: 10.1101/gr.280278.124 (PMC13431173; doi:10.1101/gr.280278.124)
Supplement: Supplement 10 [file Supplemental_Table_S3.pdf]

Supplementary Table S3. Embedding vector generation, segment split for training, testing, and validating steps.

**Genotype Embedding Model Process**	**Segments (Chromosome 8)**
Training	0, 1, 2, 3, 4, 5, 6, 8, 9, 11, 12, 13, 15, 16, 17, 19, 21, 22, 23, 27, 28, 30, 31, 32, 33, 34, 35, 36, 37, 38, 39, 40, 41, 42, 43, 44, 45, 46, 47, 49, 50, 51, 52, 53, 54, 55, 56, 57, 59, 61, 63, 64, 65, 67, 68, 69, 70, 71, 73, 75, 76, 77
Testing	14, 18, 20, 26, 29, 62, 72, 74
Validating	7, 10, 24, 25, 48, 58, 60, 66
